# Inequity in inpatient services utilization: a longitudinal comparative analysis of middle-aged and elderly patients with the chronic non-communicable diseases in China

**DOI:** 10.1186/s12939-019-1117-9

**Published:** 2020-01-06

**Authors:** Xian-zhi Fu, Lian-ke Wang, Chang-qing Sun, Dong-dong Wang, Jun-jian He, Qi-xin Tang, Qian-yu Zhou

**Affiliations:** 1 School of Political Science and Public Administration, Wuhan University, Wuhan, 430072 Hubei China; 2 Department of Social Medicine and Health Management, College of Public Health, Zhengzhou University, Zhengzhou, 450001 Henan China; 3 College of Nursing, Xinxiang Medical University, Xinxiang, 453003 Henan China

**Keywords:** Horizontal inequity, Concentration index, Health services utilization

## Abstract

**Background:**

Aging and the chronic non-communicable diseases (NCDs) challenge the Chinese government in the process of providing hospitalization services fairly and reasonably. The Chinese government has developed the basic medical insurance system to solve the problem of “expensive medical cost and difficult medical services” for vulnerable groups and alleviate the unfair phenomenon. However, few studies have confirmed its effect through longitudinal comparison. This study aimed to explore the trend in the inequity of inpatient use among middle-aged and elderly individuals with NCDs in China.

**Methods:**

This longitudinal comparative study was based on CHARLS data in 2011, 2013 and 2015. Concentration index (CI) was used to measure the variation trend of inequity of inpatient services utilization, while the decomposition method of the CI was applied to measure the factors contributing to inequity in inpatient services utilization. The effect of each factor on the change of inequity in inpatient services utilization was divided into the change of the elasticity and the change of inequality using the Oaxaca-type decomposition method.

**Results:**

The affluent middle-aged and elderly patients with NCDs used more inpatient services than poor groups. The per capita household consumption expenditure (PCE) and Urban Employee Basic Medical Insurance (UEBMI) contributed to the decline in pro-rich inequality of inpatient use, while the New Rural Cooperative Medical Scheme (NRCMS) contributed to the decline in pro-poor inequality of inpatient use.

**Conclusions:**

There was a certain degree of pro-rich unfairness in the probability and frequency of inpatient services utilization for middle-aged and elderly individuals with NCDs in China. The decrease of pro-wealth contribution of PCE and UEBMI offset the decrease of pro-poor contribution of NRCMS, and improved the equity of inpatient services utilization, favoring poor people.

## Background

It is well known that inequalities in the use of health services are widespread [1–5]. This situation may become more and more severe with the development of population aging process, which is caused by the alteration of the diseases spectrum accompanied by the aging of the population. Due to the gradual transition of diseases spectrum from infectious diseases to chronic non-communicable diseases (NCDs) [6], along with the characteristics of NCDs such as long treatment duration and high treatment costs, the middle-aged and elderly people will undoubtedly face a heavy economic burden from NCDs.

Just as many other countries in the world, equality in access to health care is a core objective of health system in China [7–9]. However, China is a developing country with the serious situation of aging population and the high prevalence of NCDs. According to statistics, from 2011 to 2017, the number of people aged over 65 in China increased from 122.88 million to 158.31 million, while the proportion of individuals aged over 65 increased from 9.1 to 11.4% [10, 11]. The analysis report of CHARLS data showed that the self-reported prevalence rate of NCDs over the age of 60 reached 78.9% in 2015 [12]. The fifth national health service survey (NHSS) of China, conducted in 2013, showed the proportion of people aged 45 to 54, 55 to 64 and 65 or above who should be hospitalized but were not hospitalized was 23.1, 19.7 and 17.7%, respectively, all higher than the national average level (17.1%) [13]. These situations have laid hidden dangers for the fair and reasonable use of inpatient services. Therefore, it is necessary and urgent to pay close attention to the inequity of inpatient services utilization in the middle-aged and elderly patients with NCDs.

Furthermore, several studies have demonstrated that the unfairness of inpatient services utilization is even higher than that of outpatient [14, 15]. The reason for this result may be that the price of inpatient services is much higher than the price of outpatient services, and it is easier to bring low-income residents into poverty, the so-called “poverty caused by illness”. Therefore, the inequity in inpatient services utilization deserves more attention than that in outpatient services utilization.

The Chinese health system has been working to address the widely acknowledged inequity in inpatient services utilization through the implementation of relevant policies. In 1998, the Urban Employee Basic Medical Insurance (UEBMI) was established to coverage individuals who were tied to formal employment status [16]. In 2002, the New Rural Cooperative Medical Scheme (NRCMS) was piloted nationwide, which provided policy benefits for rural residents to use inpatient services [17]. In 2007, the Urban Residents Basic Medical Insurance (URBMI) covered ordinary urban residents (e.g. students, children, the elderly, and unemployed urban residents) [18]. In the context of the rapid development of the national economy, most families are able to afford the small amount of outpatient services expenses. However, the impact of the basic medical insurance system on balancing the utilization of outpatient services is relatively limited. On the contrary, the main benefits of the basic medical insurance systems are aimed at the reimbursement of inpatient service cost, and alleviate the problem of “expensive medical cost and difficult medical services” for inpatients.

Many cross-sectional studies have been conducted on the inequality of health services utilization around the world. As Gao showed, among urban elderly people in China, women, low-income, and uninsured people were vulnerable groups in the process of seeking hospitalization services [19]. Crespo-Cebada (2012) found that there were pro-poor inequalities in the probability and frequency of general practitioner (GP) services utilization in Spain, mainly due to the unequal distribution of need factors [20]. Zhou (2016) identified that the UEBMI and URBMI both effectively alleviated the pro-rich inequality of inpatient services utilization in Shaanxi province of China. Chen (2018) estimated that in terms of the fairness of outpatient services, UEBMI and URBMI had an advantage over NRCMS, and in terms of hospitalization fairness, NRCMS was superior to URBMI and UEBMI [21]. Baeten (2018) verified that (the growth in) voluntary and occupational health insurance may be one of the reasons for the inequalities in access to healthcare in European 35 countries [22]. As Ataquba (2019) showed, the socio-economic inequality in health service visits needed to account for seasonal and geographical variations [23]. However, there is still a lack of longitudinal comparative studies on the inequity of hospitalization services for middle-aged and elderly individuals. In addition, we believe that the unfairness of inpatient use in middle-aged and elderly populations may be underestimated if the presence of NCDs in the population are not taken into consideration. The underlying reason is that patients with NCDs may use inpatient services frequently for a long periods of time. Therefore, a longitudinal comparative analysis of the inequity of inpatient services for middle-aged and elderly patients with NCDs using nationwide data goes beyond previous studies.

This study aims to explore the changing trend in the unfairness of probability and frequency of inpatient services utilization among middle-aged and elderly individuals with NCDs in China, and to analyze the main factors leading to this trend, and to propose feasible recommendations based on current trends.

## Methods

### Data and variable

The data source of this study was an open access database, the China Health and Retirement Longitudinal Survey (CHARLS), which was performed by the National School of Development at Peking University (NSD) and had conducted three waves of surveys in 2011, 2013 and 2015, respectively.

In this survey, multistage probability-proportional-to-scale (PPS) sampling method was adopted to select interviewees from 28 provincial administrative units in China, and interviewees’ information in the form of one-to-one questionnaire interview was collected. The questionnaire covered demographic characteristics, socioeconomic status, social security level and physical health status of the middle-aged and elderly people. Additional detailed information about this data could be found at http://charls.pku.edu.cn/en-us/page/about/charls [12].

The survey included 17,705, 18,605, and 21,095 respondents in three waves of surveys in 2011, 2013, and 2015, respectively. According to the definition of middle-aged and elderly in the published literatures, individuals aged 45 or above were included in this study [24–26]. Therefore, the inclusion criteria of samples follows below: (1) aged 45 or above; (2) completed personal information; and (3) suffered from NCDs (e.g., hypertension, dyslipidemia, diabetes or high blood sugar, chronic lung disease, cancer or malignant tumor, liver disease, heart attack, stomach or other digestive disease, emotional nervousness or psychiatric problems, memory-related disease, asthma, stroke, arthritis or rheumatism, and kidney disease). Based on these criteria conditions, in the data of three waves of the survey in 2011, 2013 and 2015, the sample size of our final analysis was 107,24, 8005 and 10,651, respectively.

Dependent variables for this study included two aspects: (1) Did the respondent use the inpatient services at least once in the past year? (2) How many times did respondents use inpatient services in the past year? The former’s answer was “yes” or “no”, while the latter’s answer was “0, 1, 2, 3, 4 or more.”

Independent variables were divided into three components (predisposing variables, enabling variables and need variables), in accordance with the Andersen’s behavioral model [27]. Since its inception, the Andersen’s model had been verified by a number of empirical studies and was widely considered to be the most appropriate model for analyzing the utilization of health services [28, 29].

The predisposing variables were the individual characteristics of people who tend to use inpatient services before the onset of the diseases [27, 30, 31], including gender (female, male), age (45~59, 60~74, 75+), education level (illiterate, primary school, middle school, high school and above), marital status (married, unmarried) and employment status (unemployed, agricultural work, working in units, other working).

The enabling variables referred to an individual’s ability to access inpatient services [27, 30, 31], including health insurance schemes (no health insurance, UEBMI, URBMI, NRCMS, other health insurance), geographic location (east, central, west), and residency location (urban, rural). The per capita household consumption expenditure (PCE), as an indicator to measure family economic level, was closely related to well-being and easy to calculate [15]. Therefore, this study classified PCE as the enabling variables.

The need variables represented the perceived needs and actual needs of the inpatient services utilizaiton [27, 30, 31], including self-assessed health status, disability, disability in physical activity of daily living (PADL) and disability in instrumental activity of daily living (IADL). Self-assessed health status was divided into “healthy” or “unhealthy”, while disability, PADL and IADL were all categorized as “yes” or “no”.

With regard to disabilities, this study defined it as any of the following six categories: physical disabilities, brain damage, vision disorder, hearing disorder and speech impediment. According to the Activity of Daily Living Scale developed by Lawton and Brody in 1969 [32], this study divided the activity of daily living (ADL) into PADL (dressing, showering, eating, getting into or out of bed, using the toilet, controlling urination and defecation) and IADL (doing household chores, preparing hot meals, shopping, managing assets, taking medications). In each of the 11 daily activities listed above, if the respondents failed to finish any of them independently, disability in PADL or IADL could be defined.

### Methodology

In this paper, the concentration index (CI) proposed by Wagstaff and Van Doorslaer was applied to measure the inequality of inpatient services utilization [33, 34]. The CI is sensitive to the population distribution of socioeconomic groups, and more accurate while expressing the fairness of inpatient service utilization under different socioeconomic levels. Apart from this, the CI is applicable to time trend research [35, 36]. The concentration curve plots the cumulative proportion of the middle-aged and elderly, which is ranked by PCE from the poorest to richest (x-axis), against the cumulative proportion of the inpatient services utilization (y-axis), while the CI is twice the area enclosed by the concentration curve and absolute fairness line [33, 37]. The maximum and minimum of the CI range from − 1 to + 1 [36]. The smaller the absolute value is, the more fair it represents. When the CI is zero, the distribution of inpatient service utilization is absolutely fair [35]. A positive CI indicates that the distribution of inpatient service utilization is more conducive to the rich, and a negative CI indicates that the distribution of inpatient service utilization is more favorable to the poor.

The CI is calculated by the following eq. (1):

$$ \mathrm{CI}=\frac{2}{\upmu}{\operatorname{cov}}_w\left({\mathrm{y}}_i,{\mathrm{r}}_i\right)\kern8.5em (1) $$

As shown in equation (1), y_*i*_ represents the relevant indicators for the inpatient services utilization. r_*i*_ is the fractional rank of the respondents in terms of PCE and μ is the mean of inpatient services utilization.

The method of decomposition of CI, proposed by Wagstaff [33], can be used to analyze the contribution of relevant influencing factors to the CI in the study of the inequality of inpatient services utilization. In order to decompose the CI, the regression model established in this study is shown in equation (2):

$$ {\mathrm{y}}_i=\updelta +{\sum}_k{\upgamma}_{\mathrm{k}}{\mathrm{z}}_{ki}+{\upvarepsilon}_i\kern9.25em (2) $$

In the equation (2), z_*k*_ represents the independent variables, *i* is the sample size, and δ, γ_k_ and ε represent the constant term, marginal effect and error term, respectively.

The model of decomposition of CI is shown in equation (3):

$$ \mathrm{CI}={\sum}_{\mathrm{k}}\frac{\upgamma_{\mathrm{k}}{\overline{\mathrm{z}}}_{\mathrm{k}}}{\upmu}{\mathrm{C}}_{\mathrm{k}}+\frac{GC_{\varepsilon }}{\upmu}\kern9em (3) $$

Where γ_k_ is the marginal effect of independent variables. ${\overline{\mathrm{z}}}_{\mathrm{k}}$ represents the mean of independent variables. C_k_ represents the CI of independent variables, and (*GC*_*ε*_/μ) represents the error term [38].

In order to eliminate the interference of need variables (including self-assessed health status, disability, PADL and IADL), this study also introduces the horizontal inequity index (HI) to measure the inequality of the inpatient services utilization for middle-aged and elderly patients with NCDs in China. HI can be calculated by substracting the contribution of need variables from CI of inpatient services utilization [33], as shown in equation (4):

$$ \mathrm{HI}=\mathrm{CI}-{\sum}_{\mathrm{k}}\left(\frac{\upgamma_{\mathrm{k}}{\overline{\mathrm{z}}}_{\mathrm{k}}}{\upmu}\right){\mathrm{C}}_{\mathrm{k}}\kern9.75em (4) $$

Similar to the CI, a positive HI indicates that the distribution of inpatient service utilization is more conducive to the affluent population and vice versa.

In order to measure the contribution of relevant influencing factors to the change of inequality in inpatient services utilization, the method of Oaxaca-type decomposition is applied [39], as shown in equation (5):

$$ \triangle \mathrm{CI}={\sum}_{\mathrm{k}}{\upeta}_{\mathrm{k}\mathrm{t}}\left({\mathrm{C}}_{\mathrm{k}\mathrm{t}}-{\mathrm{C}}_{\mathrm{k}\mathrm{t}-1}\right)+{\sum}_{\mathrm{k}}{\mathrm{C}}_{\mathrm{k}\mathrm{t}-1}\left({\upeta}_{\mathrm{k}\mathrm{t}}-{\upeta}_{\mathrm{k}\mathrm{t}-1}\right)+\triangle \left(\frac{GC_{\varepsilon }}{\upmu}\right)\kern0.5em (5) $$

Where k, t and △ represent the total number of independent variables, time period and first differences. η is elasticity of CI, calculated as $\left({\upgamma}_k{\overline{\mathrm{x}}}_{\mathrm{k}}/\upmu \right)$.

In this study, the probit model and the general negative binomial regression model were used to conduct regression analysis on the probability and frequency of inpatient services utilization, respectively, and STATA software version 15.1 was used for all the analyses.

## Results

### Descriptive statistics

Table 1 reports the social demographic characteristics of the respondents.From 2011 to 2015, the probability and frequency of inpatient services utilization of the middle-aged and elderly respondents with NCDs showed a mild growth trend and the percentage of women was steadily greater than 50%. The participants aged 45 to 59 fell by 9.12 percentage points, while the individuals aged 60 to 74 rose by 8.91 percentage points. Illiterate participants decreased by 2.14 percentage points, while the respondents with education level of primary school, middle school, high school or above increased slightly. The percentage of middle-aged and elderly individuals without jobs and engaged in agriculture was higher, while the ratio of the middle-aged and elderly patients working in units fell by 4.34 percentage points in the year of 2011 to 2015. From 2011 to 2013, the proportion of the insured population covered by UEBMI, URBMI and NRCMS all revealed an upward trend, and then appeared a downward trend over the year from 2013 to 2015. The ratio of the population categoried by geographical location could be discovered: west > central > east. PCE increased significantly, from 8368.71 YUAN in 2011 to 16,063.40 YUAN in 2015. Compared with 2011, the healthy and disabled middle-aged and elderly individuals increased by 4.69 percentage points and 15.99 percentage points in 2015 respectively. From 2011 to 2013, middle-aged and elderly patients with disability of PADL and IADL decreased by 0.5 percentage points and 2.89 percentage points, respectively, and then increased by 3.39 percentage points and 2.48 percentage points, respectively in the year of 2013 to 2015. Married respondents and urban respondents had relatively stable proportion.

**Table 1:** Social demographic characteristics, China, 2011–2015

Variables	Description	2011 (All = 10,724)	2013 (All = 8005)	2015 (All = 10,651)
Dependent variables	Probability of inpatient service utilization (Percentage)	12.02	16.31	17.28
	Frequency of inpatient services utilization (Means)	0.17	0.25	0.26
Predisposing variables				
Gender	Female^1^, n(%)	5603(52.25)	4279 (53.45)	5653 (53.07)
	Male, n(%)	5121 (47.75)	3726 (46.55)	4998 (46.93)
Age	45~59^1^, n(%)	5551 (51.76)	3685 (46.03)	4542 (42.64)
	60~74, n(%)	4188 (39.05)	3614 (45.15)	5108 (47.96)
	75+, n(%)	985 (9.19)	706 (8.82)	1001 (9.40)
Education level	Illiterate^1^, n(%)	5030 (46.90)	3703 (46.26)	4767 (44.76)
	Primary school, n(%)	2332 (21.75)	1748 (21.84)	2392 (22.46)
	Middle school, n(%)	2107 (19.65)	1624 (20.29)	2184 (20.51)
	High school and above, n(%)	1255 (11.70)	930 (11.62)	1308 (12.28)
Marital status	Married, n(%)	9322 (86.93)	7024 (87.75)	9254 (86.88)
	Unmarried^1^, n(%)	1402 (13.07)	981 (12.25)	1397 (13.12)
Employment status	Unemployed^1^, n(%)	4397 (41.00)	3027 (37.81)	4142 (38.89)
	Agricultural work, n(%)	4027 (37.55)	3397 (42.44)	3921 (36.81)
	Working in units, n(%)	1429 (13.33)	908 (11.34)	958 (8.99)
	Other working, n(%)	871 (8.12)	673 (8.41)	1630 (15.30)
Enabling variables				
Health insurance schemes	No health insurance^1^, n(%)	651 (6.07)	216 (2.70)	729 (6.84)
	UEBMI, n(%)	1091 (10.17)	918 (11.47)	1064 (9.99)
	URBMI, n(%)	482 (4.49)	396 (4.95)	438 (4.11)
	NRCMS, n(%)	7732 (72.10)	5902 (73.73)	7000 (65.72)
	Other health insurance, n(%)	768 (7.16)	573 (7.16)	1420 (13.33)
Geographic location	East^1^, n(%)	3276 (30.55)	2452 (30.63)	3394 (31.87)
	Central, n(%)	3704 (34.54)	2634 (32.90)	3533 (33.17)
	West, n(%)	3744 (34.91)	2919 (36.46)	3724 (34.96)
Residency location	Urban, n(%)	4201 (39.17)	3055 (38.16)	4165 (39.10)
	Rural^1^, n(%)	6523 (60.83)	4950 (61.84)	6486 (60.90)
PCE	Mean (RMB)	8368.71	12,116.83	16,063.40
Need variables				
Self-assessed health status	Healthy, n(%)	6632 (61.84)	5281 (65.97)	7086 (66.53)
	Unhealthy^1^, n(%)	4092 (38.16)	2724 (34.03)	3565 (33.47)
Disability	Yes, n(%)	2223 (20.73)	2338 (29.21)	3911 (36.72)
	No^1^, n(%)	8501 (79.27)	5667 (70.79)	6740 (63.28)
PADL	Yes, n(%)	2290 (21.35)	1669 (20.85)	2582 (24.24)
	No^1^, n(%)	8434 (78.65)	6336 (79.15)	8069 (75.76)
IADL	Yes, n(%)	2798 (26.09)	1857 (23.20)	2735 (25.68)
	No^1^, n(%)	7926 (73.91)	6148 (76.80)	7916 (74.32)

Note: ^1^Reference group; *PCE* per capita household consumption expenditure; *PADL* Physical Activity of Daily Living; *IADL* Instrumental Activity of Daily Living; *UEBMI* Urban Employee Basic Medical Insurance; *NRCMS* New Rural Cooperative Medical Scheme; *URBMI* Urban Residents Basic Medical Insurance

### Inequality and horizontal inequity for inpatient services utilization

**Fig. 1:**
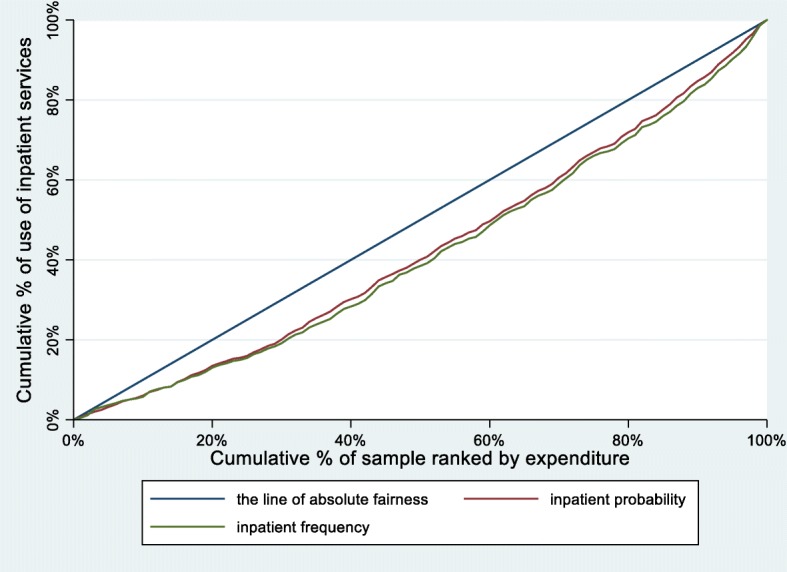
Concentration curves for use of inpatient services, China, 2011

**Fig. 2:**
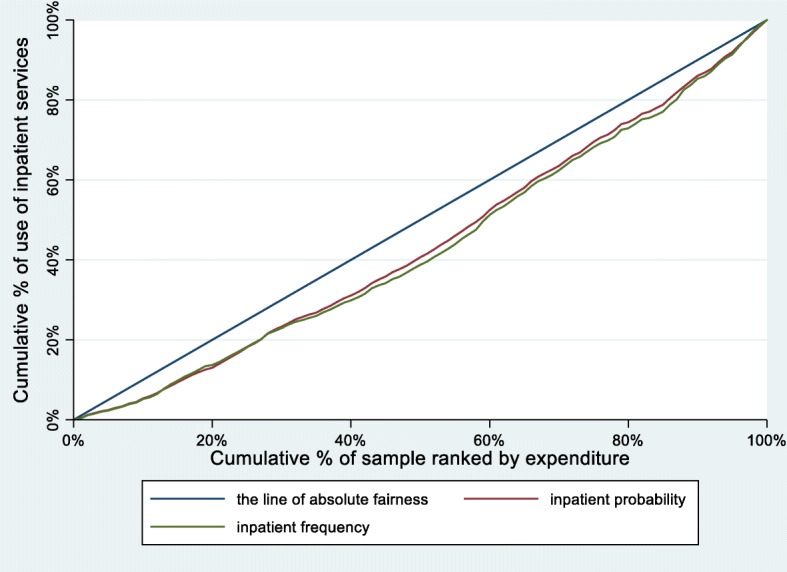
Concentration curves for use of inpatient services, China, 2013

**Fig. 3:**
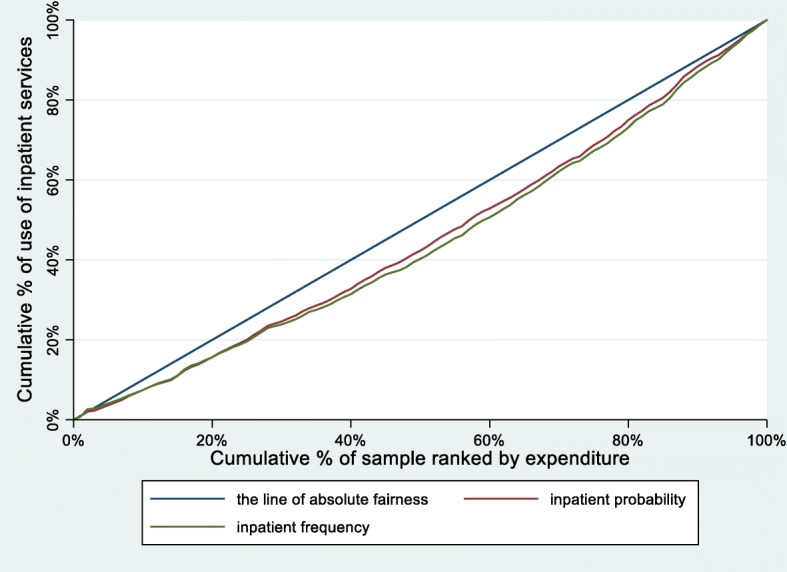
Concentration curves for use of inpatient services, China, 2015

Table 2 illustrates the CIs and the HIs for probability and number of inpatient services utilization from 2011 to 2015. The corresponding concentration curves of probability and frequency of inpatient services utilization are also depicted in Fig. 1, Fig. 2 and Fig. 3.

**Table 2:** Inequality and horizontal Inequity for inpatient services utilization, China, 2011–2015

Variable	Probability of Inpatient Visits		Frequency of Inpatient Visits	
	CI	HI	CI	HI
2011	0.1479	0.1708	0.1695	0.1970
2013	0.1228	0.1417	0.1391	0.1598
2015	0.0979	0.1166	0.1185	0.1410

From 2011 to 2015, the CIs and HIs of the probability of inpatient services utilization decreased by 33.81 and 31.73%, respectively. The concentration curves of probability of inpatient services utilization were all below the line of absolute fairness. The area between the concentration curves of probability of inpatient services utilization and the line of absolute fairness also showed a steady trend of decreasing.

Similar to the probability of inpatient services utilization, the CIs and HIs of the number of inpatient services utilization decreased by 30.09 and 28.43%, respectively. The concentration curves of number of inpatient services utilization were parallel to the probability of inpatient services utilization.

### Decomposition of inequality in inpatient services utilization

**Fig. 4:**
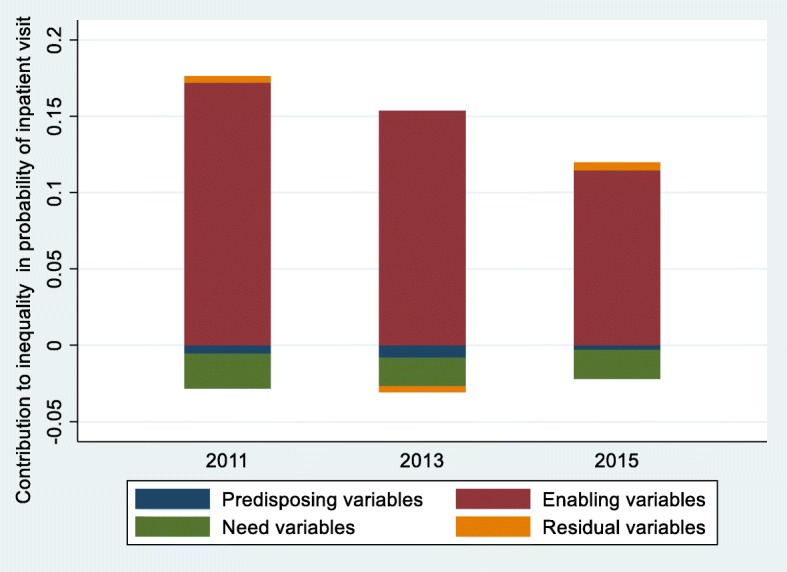
Decomposition of inequality in probability of inpatient services utilization, China, 2011–2015

After decomposing the CIs of the probability of inpatient services utilization, the contribution of related variables to the overall inequality is shown in Table 3. The contribution of three variable-groups is shown in Fig. 4.

**Table 3:** Contribution to inequalities in probability of inpatient visits, China, 2011–2015

Variable	2011		2013		2015	
	ME^2^	Cont^3^	ME^2^	Cont^3^	ME^2^	Cont^3^
Predisposing variables		−0.0055		−0.0080		−0.0031
Gender						
Female^1^						
Male	0.0201**	0.0010	0.0257**	0.0012	0.0061	0.0003
Age						
45~59^1^						
60~74	0.0281**	−0.0035	0.0354**	− 0.0048	0.0372**	− 0.0047
75+	0.0507**	− 0.0035	0.0666**	− 0.0032	0.0671**	−0.0044
Education level						
Illiterate^1^						
Primary school	−0.0108	0.0004	−0.0015	0.0000	0.0077	− 0.0002
Middle school	−0.0001	0.0000	−0.0177	− 0.0022	0.0190	0.0016
High school and above	−0.0143	− 0.0047	− 0.0100	−0.0021	0.0000	0.0000
Marital status						
Unmarried^1^						
Married	0.0229**	0.0019	0.0043	0.0002	0.0019	0.0001
Employment status						
Unemployed^1^						
Agricultural work	−0.0287**	0.0142	−0.0325**	0.0077	−0.0495**	0.0107
Working in units	−0.0448**	− 0.0069	− 0.0418**	−0.0024	− 0.0587**	−0.0041
Other working	−0.0503**	−0.0044	− 0.0396**	−0.0024	− 0.0525**	−0.0024
Enabling variables		0.1719		0.1536		0.1147
Health insurance schemes						
No health insurance^1^						
UEBMI	− 0.1088**	0.0432	0.0923**	0.0213	0.0849**	0.0164
URBMI	0.0833**	0.0067	0.0005	0.0000	0.0466	0.0009
NRCMS	0.0599**	−0.0359	0.0583*	− 0.0200	0.0402**	− 0.0121
Other health insurance	0.0992**	0.0150	0.0746*	0.0064	0.0647**	0.0071
Geographic location						
East^1^						
Central	0.0212**	−0.0021	0.0356**	−0.0016	0.0166	−0.0006
West	0.0463**	− 0.0046	0.0604**	0.0004	0.0501**	0.0005
Residency location						
Rural^1^						
Urban	0.0111	0.0077	0.0220*	0.0072	0.0062	0.0017
PCE	0.0339**	0.1419	0.0435**	0.1399	0.0320**	0.1008
Need variables		−0.0229		− 0.0188		− 0.0189
Self-assessed health status						
Unhealthy^1^						
Healthy	−0.0849**	−0.0135	− 0.1443**	−0.0145	− 0.1381**	−0.0099
Disability						
No^1^						
Yes	−0.0024	0.0005	0.0161	−0.0017	0.0123	−0.0020
PADL						
No^1^						
Yes	0.0212**	−0.0034	0.0106	−0.0009	0.0494**	−0.0043
IADL						
No^1^						
Yes	0.0323**	−0.0065	0.0186	−0.0017	0.0304**	−0.0027
Residual variables		0.0044		−0.0040		0.0052

Note: * *p* < 0.05; ** *p* < 0.01; ^1^Reference group; ^2^Marginal effect; ^3^Contribution; *PCE* logarithm value of the per capita household expenditure; *PADL* Physical Activity of Daily Living; *IADL* Instrumental Activity of Daily Living; *UEBMI* Urban Employee Basic Medical Insurance; *NRCMS* New Rural Cooperative Medical Scheme; *URBMI* Urban Residents Basic Medical Insurance

During the period from 2011 to 2015, the contribution of enabling variables to inequality of probability of inpatient services utilization was the highest, followed by the need variables and the predisposing variables was the least. Among the enabling variables, the top three contributing factors were PCE, UEBMI and NRCMS. PCE made the greatest pro-rich contribution to the inequality of probability of inpatient use (0.1419, 0.1399 and 0.1008, respectively). The decomposition result of the CI of UEBMI had a pro-wealth impact on probability of inpatient use, which decreased steadily from 0.0432 in 2011 to 0.0164 in 2015. The pro-poor contribution of NRCMS had a gradually decreasing trend, from − 0.0359 in 2011 to − 0.0121 in 2015, meanwhile, the changing trend of need variables was similar to NRCMS, from − 0.0229 in 2011 to − 0.0189 in 2015. Compared with the other two variable-groups, the predisposing variables had the least contribution to unfairness of the probability of inpatient use. Other detailed data on the decomposition of inequality in probability of inpatient services utilization is shown in Additional file 1: Tables S1, S2 and S3.

**Fig. 5:**
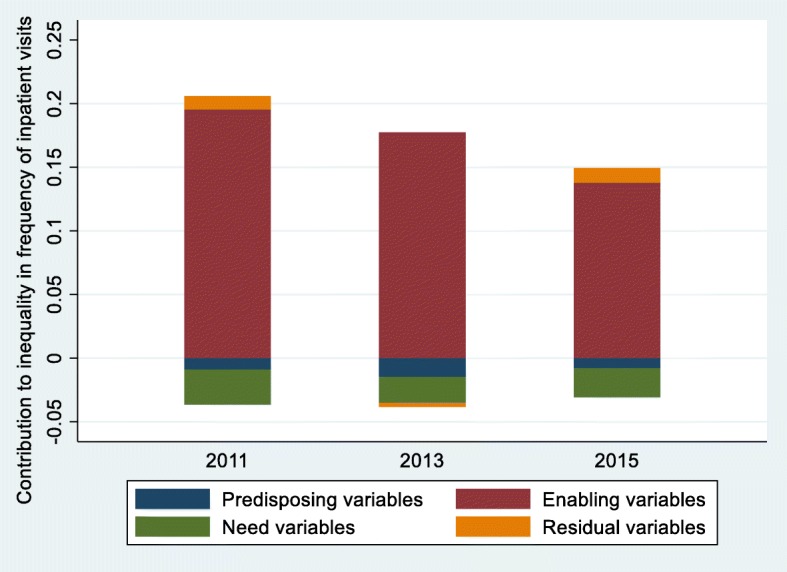
Decomposition of inequality in frequency of inpatient services utilization, China, 2011–2015

Table 4 displays the contribution for each variable to inequality of the frequency of inpatient services utilization. The contribution of three variable-groups is shown in Fig. 5.

**Table 4:** Contribution to inequalities of frequency of inpatient visits, China, 2011–2015

Variable	2011		2013		2015	
	ME^2^	Cont^3^	ME^2^	Cont^3^	ME^2^	Cont^3^
Predisposing variables		−0.0090		− 0.0148		− 0.0081
Gender						
Female^1^						
Male	0.0428**	0.0015	0.0581**	0.0017	0.0058	0.0002
Age						
45~59^1^						
60~74	0.0492**	−0.0042	0.0536**	−0.0047	0.0643**	−0.0053
75+	0.0916**	−0.0043	0.1075**	−0.0034	0.1316**	−0.0056
Education level						
Illiterate^1^						
Primary school	−0.0229	0.0005	− 0.0016	0.0000	0.0039	−0.0001
Middle school	−0.0142	− 0.0020	− 0.0470	− 0.0038	0.0280	0.0015
High school and above	−0.0290	− 0.0065	− 0.0914**	− 0.0121	− 0.0172	−0.0024
Marital status						
Unmarried^1^						
Married	0.0338*	0.0019	0.0047	0.0002	0.0181	0.0004
Employment status						
Unemployed^1^						
Agricultural work	−0.0630**	0.0216	− 0.0980**	0.0150	− 0.1035**	0.0146
Working in units	−0.1070**	− 0.0114	− 0.1191**	− 0.0044	− 0.1717**	−0.0078
Other working	−0.0999**	−0.0061	− 0.0849**	− 0.0033	− 0.1189**	− 0.0036
Enabling variables		0.1954		0.1775		0.1379
Health insurance schemes						
No health insurance^1^						
UEBMI	0.1202**	0.0330	0.1721**	0.0256	0.1115**	0.0141
URBMI	0.0902*	0.0050	0.0693	0.0016	0.0765	0.0009
NRCMS	0.0875*	−0.0362	0.1433**	−0.0316	0.0475	−0.0093
Other health insurance	0.1204**	0.0126	0.1625**	0.0090	0.1076**	0.0077
Geographic location						
East^1^						
Central	0.0465**	−0.0032	0.0974**	− 0.0028	0.0322	−0.0007
West	0.0899**	−0.0062	0.1370**	0.0006	0.0917**	0.0006
Residency location						
Rural^1^						
Urban	0.0064	0.0031	0.0299	0.0063	0.0013	0.0002
PCE	0.0647**	0.1873	0.0816**	0.1688	0.0605**	0.1244
Need variables		−0.0275		−0.0206		− 0.0227
Self-assessed health status						
Unhealthy^1^						
Healthy	−0.1537**	−0.0169	− 0.2319**	− 0.0150	− 0.2533**	−0.0118
Disability						
No^1^						
Yes	−0.0082	0.0011	0.0309	−0.0021	0.0253	−0.0026
PADL						
No^1^						
Yes	0.0375**	−0.0042	0.0339	−0.0019	0.0933**	−0.0054
IADL						
No^1^						
Yes	0.0534**	−0.0075	0.0276	−0.0016	0.0499**	−0.0029
Residual variables		0.0106		−0.0030		0.0113

Note: * p < 0.05; ** p < 0.01; ^1^ Reference group; ^2^Marginal effect; ^3^Contribution; *PCE* logarithm value of the per capita household expenditure; *PADL* Physical Activity of Daily Living; *IADL* Instrumental Activity of Daily Living; *UEBMI* Urban Employee Basic Medical Insurance; *NRCMS* New Rural Cooperative Medical Scheme; *URBMI* Urban Residents Basic Medical Insurance

During 2011 to 2015, the order of three variable-groups which contribute to the unfairness of the frequency of inpatient service utilization was enabling variables, need variables and predisposing variables, respectively. In the enabling variables, the three variables with larger decomposition value of the CI were PCE, NRCMS and UEBMI in turn. PCE made the greatest pro-wealth contribution to the inequality of frequency of inpatient use in the year of 2011 to 2015 and its contribution value showed a steady trend of decline (from 0.1873 to 0.1244). The decomposition result of the CI of UEBMI presented a pro-rich contribution, from 0.0330 in 2011 to 0.0141 in 2015. On the contrary, NRCMS contributed as high as − 0.0362, − 0.0316 and − 0.0093 to the inequality in frequency of inpatient use in the year of 2011, 2013 and 2015. Similar to the NRCMS, the need variables had negative contribution to inequality in the number of inpatient service utilization. In addition, the predisposing variables provided relatively small contribution to the inequality. Other detailed statistical analysis data is shown in Additional file 1: Tables S4, S5 and S6.

### Change in the decomposition of the concentration index

**Fig. 6:**
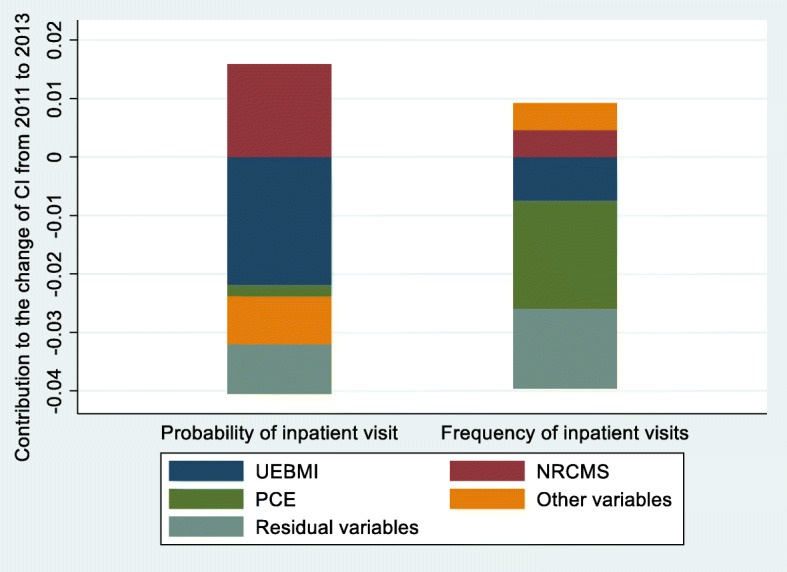
Change in the decomposition of the concentration index, China, 2011–2013

**Fig. 7:**
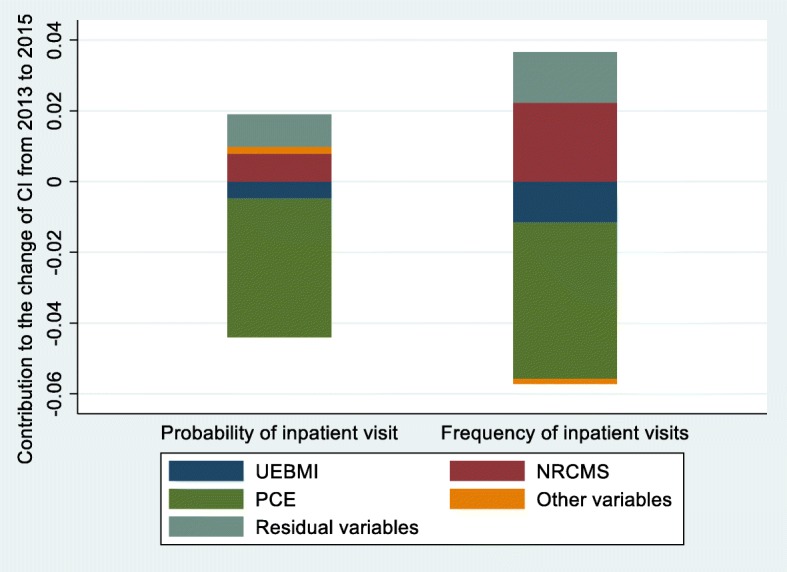
Change in the decomposition of the concentration index, China, 2013–2015

The change in the decomposition of the CI for probability and frequency of inpatient services utilization is displayed in Table 5 and Table 6. Figure 6 and Fig. 7 show the change in the contribution of the major variables.

**Table 5:** Oaxaca-type decomposition of change for inequality in probability and frequency of inpatient service utilization, China, 2011–2013

Variable	Probability of inpatient visits			Frequency of inpatient visits		
	△CI^2^	△Elast^3^	Change	△CI^2^	△Elast^3^	Change
Predisposing variables	−0.0039	0.0015	−0.0024	− 0.0057	− 0.0001	− 0.0058
Gender						
Female^1^						
Male	0.0002	−0.0001	0.0001	0.0004	−0.0001	0.0003
Age						
45~59^1^						
60~74	−0.0011	− 0.0002	− 0.0013	−0.0011	0.0006	−0.0005
75+	0.0000	0.0002	0.0002	0.0000	0.0010	0.0010
Education level						
Illiterate^1^						
Primary school	0.0000	−0.0003	−0.0003	0.0000	−0.0005	− 0.0005
Middle school	0.0005	−0.0027	−0.0022	0.0008	−0.0026	− 0.0018
High school and above	0.0003	0.0023	0.0026	0.0019	−0.0075	−0.0056
Marital status						
Unmarried^1^						
Married	0.0000	−0.0016	−0.0016	0.0000	−0.0018	− 0.0018
Employment status						
Unemployed^1^						
Agricultural work	−0.0057	− 0.0008	− 0.0065	−0.0111	0.0044	−0.0067
Working in units	0.0017	0.0029	0.0046	0.0030	0.0040	0.0070
Other working	0.0003	0.0018	0.0021	0.0004	0.0024	0.0028
Enabling variables	−0.0023	− 0.0160	− 0.0183	0.0008	− 0.0189	− 0.0181
Health insurance schemes						
No health insurance^1^						
UEBMI	−0.0091	− 0.0128	− 0.0219	−0.0110	0.0035	−0.0075
URBMI	0.0000	−0.0067	− 0.0067	− 0.0013	− 0.0021	− 0.0034
NRCMS	0.0063	0.0096	0.0159	0.0100	−0.0054	0.0046
Other health insurance	−0.0019	− 0.0067	− 0.0086	− 0.0026	− 0.0009	−0.0035
Geographic location						
East^1^						
Central	0.0009	−0.0004	0.0005	0.0015	−0.0012	0.0003
West	0.0050	0.0000	0.0050	0.0074	−0.0006	0.0068
Residency location						
Rural^1^						
Urban	−0.0037	0.0032	−0.0005	− 0.0033	0.0065	0.0032
PCE	0.0002	−0.0021	−0.0019	0.0001	−0.0186	− 0.0185
Need variables	0.0063	−0.0021	0.0042	0.0071	−0.0002	0.0069
Self-assessed health status						
Unhealthy^1^						
Healthy	0.0035	−0.0045	− 0.0010	0.0037	− 0.0018	0.0019
Disability						
No^1^						
Yes	0.0017	−0.0038	−0.0021	0.0021	−0.0052	− 0.0031
PADL						
No^1^						
Yes	0.0003	0.0022	0.0025	0.0007	0.0016	0.0023
IADL						
No^1^						
Yes	0.0008	0.0041	0.0049	0.0008	0.0051	0.0059
Residual variables			−0.0084			− 0.0136

Note: ^1^Reference group; ^2^Elasticties_2013_*(C_2013_-C_2011_); ^3^C_2011_*(Elasticties_2013_-Elasticties_2011_); *PCE* logarithm value of the per capita household expenditure; *PADL* Physical Activity of Daily Living; *IADL* Instrumental Activity of Daily Living; *UEBMI* Urban Employee Basic Medical Insurance; *NRCMS* New Rural Cooperative Medical Scheme; *URBMI* Urban Residents Basic Medical Insurance

**Table 6:** Oaxaca-type decomposition of change for inequality in probability and frequency of inpatient service utilization, China, 2013–2015

Variable	Probability of inpatient visits			Frequency of inpatient visits		
	△CI^2^	△Elast^3^	Change	△CI^2^	△Elast^3^	Change
Predisposing variables	0.0010	0.0039	0.0049	0.0010	0.0058	0.0068
Gender						
Female^1^						
Male	0.0000	−0.0009	− 0.0009	0.0000	− 0.0015	− 0.0015
Age						
45~59^1^						
60~74	0.0004	−0.0003	0.0001	0.0004	−0.0010	−0.0006
75+	−0.0011	0.0000	−0.0011	−0.0014	− 0.0008	−0.0022
Education level						
Illiterate^1^						
Primary school	−0.0001	−0.0001	− 0.0002	0.0000	0.0000	−0.0000
Middle school	−0.0007	0.0045	0.0038	−0.0007	0.0060	0.0053
High school and above	0.0000	0.0021	0.0021	−0.0001	0.0098	0.0097
Marital status						
Unmarried^1^						
Married	0.0000	−0.0001	−0.0001	− 0.0002	0.0004	0.0002
Employment status						
Unemployed^1^						
Agricultural work	0.0011	0.0019	0.0030	0.0015	−0.0019	−0.0004
Working in units	−0.0016	−0.0001	− 0.0017	−0.0030	− 0.0004	−0.0034
Other working	0.0030	−0.0030	0.0000	0.0044	−0.0047	−0.0003
Enabling variables	−0.0023	−0.0365	− 0.0388	−0.0019	− 0.0375	−0.0394
Health insurance schemes						
No health insurance^1^						
UEBMI	0.0004	−0.0052	−0.0048	0.0003	−0.0118	− 0.0115
URBMI	−0.0005	0.0013	0.0008	−0.0005	−0.0002	− 0.0007
NRCMS	−0.0005	0.0084	0.0079	−0.0004	0.0227	0.0223
Other health insurance	−0.0027	0.0034	0.0007	−0.0029	0.0016	−0.0013
Geographic location						
East^1^						
Central	0.0001	0.0009	0.0010	0.0002	0.0019	0.0021
West	0.0002	−0.0001	0.0001	0.0002	−0.0002	0.0000
Residency location						
Rural^1^						
Urban	−0.0003	−0.0052	− 0.0055	0.0000	− 0.0060	−0.0060
PCE	0.0008	−0.0400	−0.0392	0.0012	−0.0455	− 0.0443
Need variables	0.0033	−0.0034	−0.0001	0.0038	−0.0060	− 0.0022
Self-assessed health status						
Unhealthy^1^						
Healthy	0.0033	0.0013	0.0046	0.0040	−0.0008	0.0032
Disability						
No^1^						
Yes	−0.0005	0.0002	−0.0003	−0.0006	0.0000	−0.0006
PADL						
No^1^						
Yes	0.0003	−0.0037	−0.0034	0.0003	−0.0038	− 0.0035
IADL						
No^1^						
Yes	0.0001	−0.0012	−0.0011	0.0001	−0.0015	− 0.0014
Residual variables			0.0092			0.0143

Note: ^1^Reference group; ^2^Elasticties_2015_*(C_2015_-C_2013_); ^3^C_2013_*(Elasticties_2015_-Elasticties_2013_); *PCE* logarithm value of the per capita household expenditure; *PADL* Physical Activity of Daily Living; *IADL* Instrumental Activity of Daily Living; *UEBMI* Urban Employee Basic Medical Insurance; *NRCMS* New Rural Cooperative Medical Scheme; *URBMI* Urban Residents Basic Medical Insurance

From 2011 to 2015, enabling variables made the greatest contribution to the change of inequality for probability and frequency of inpatient services utilization. During the whole period from 2011 to 2015, PCE, UEBMI and NRCMS made great contribution to the change of inequality for probability and frequency of inpatient use, from which PCE and UEBMI contributed to the decrease of pro-rich inequality and NRCMS contributed to the decrease of pro-poor inequality.

From 2011 to 2013, for UEBMI and NRCMS, the change in inequality rather than in elasticity was dominant in the contribution to inequality in frequency of inpatient use and the change in inequality and in elasticity had reinforcing effect to change the inequality in probability of inpatient use. Moreover, during the period of 2013 to 2015, all the three main determinants had much higher change in elasticity than inequality. The other variables provided relatively minor contribution to the change of inequality, while the residual variables made great contribution to the change of inequality.

## Discussion

Through deep analysis of the CHARLS data, this research provides evidence for the inequality of inpatient services utilization for middle-aged and elderly patients with NCDs in China. Based on the CIs and HIs, we identified that there was a certain degree of pro-rich inequality in the probability and frequency of inpatient services utilization and the unfairness was gradually alleviated over time. It was consistent with the research conclusions of relevant scholars [15, 40, 41], indicating that the difference in the probability and frequency of inpatient use between the poor and the rich was gradually decreasing. In addition, we detected that the inequality of frequency of inpatient use was slightly more serious than that of the inequality of probability of inpatient use. It indicated that the inequality of frequency of inpatient use for the middle-aged and elderly with NCDs was mainly arised from the initial hospitalization rather than the re-hospitalization. It could also be interpreted that the probability of choosing the first hospitalization for the rich was significantly greater than that of the poor, but after the initial hospitalization, the probability of repeated utilization of inpatient services of the rich was only slightly greater than that of the poor.

In the analysis of influencing factors, we identified the main variables (e.g., PCE, URBMI and NRCMS) affecting the inequality of the probability and frequency of inpatient services utilization for middle-aged and elderly people in China. For both the probability and number of inpatient use, PCE contributed the greatest pro-rich inequality, which indicated that the difference in economic level was still the prime factor leading to the unfairness of inpatient use in China [14, 15, 41, 42]. From 2011 to 2015, among the respondents who should be hospitalized but were not hospitalized in the past year, the proportion of those who did not choose to be hospitalized due to “not enough money” was more than 55% and the CI of corresponding individuals was less than zero (Additional file 1: Table S7). It indicated that this situation occurred mostly in poor middle-aged and elderly patients with NCDs, and provided a concrete evidence to confirm the point mentioned.

Our study also revealed that health insurance scheme was the second most important factor affecting inequality of inpatient services utilization. The pro-wealth contribution of UEBMI was second only to that of PCE, which was quite different from the viewpoint that medical insurance alleviated the unfairness of health services utilization [43–45]. This outcome could be explained from two aspects: (1) UEBMI covered the people with formal jobs, which determined that the insured had a stable income source and stronger economic capability. (2) From 2011 to 2015, the out-of-pocket inpatient expenditure as a percentage of total inpatient expenditure for individuals covered by UEBMI was less than 35%, which was significantly lower than URBMI and NRCMS (Additional file 1:Table S7). It meant that there was no significant financial risk for insured individuals of UEBMI in inpatient use, which will easily lead to the overuse of inpatient services by affluent people.

In contrast to UEBMI, NRCMS was the most important factor to mitigate inequality in inpatient use. It had been declared by the conclusions of previous scholars [15, 40]. This study pronounced that in the formulation of NRCMS, the insured individuals were set as the rural population with poor economic capacity. This setup played a decisive role to reduced pro-rich inequality in inpatient services utilization.

The comparison between Fig. 4 and Fig. 5 shows that the contribution of three variable-groups to the inequity of probability of inpatient service utilization is similar to the number of inpatient service utilization. Enabling variables made the strong pro-rich inequality of the probability and frequency of inpatient service utilization. Need variables slightly decreased the pro-rich inequality of inpatient services utilization, and self-assessed health status was the main contributor to this outcome. Jason’s research found that the individuals with poorer economic capacity were related to poorer health status, in other words, they need more inpatient services [46]. Therefore, this was a good sign that individuals with more demand for inpatient services received more inpatient services. Additionally, as shown in Additional file 1: Table S7, the poor were more likely not to seek inpatient care due to “ not willing to go to the hospital “ in 2015. Hence, it is necessary to increase the publicity of NCDs for health hazards and to improve the health awareness of middle-aged and elderly patients with NCDs, which is conducive to alleviating the pro-rich inequity of inpatient services use.

More importantly, this research found the main factors leading to the change of inequality of inpatient use for middle-aged and elderly patients with NCDs in China through longitudinal comparison. As shown in both Fig. 6 and Fig. 7, the main contributors of the change of inequity of inpatient service utilization were PCE, NRCMS and UEBMI. It can be expressed as in spite of the pro-poor contribution of NRCMS had decreased, the reduction of the pro-rich contribution of PCE and UEBMI offset it, and generally alleviated the pro-rich inequality of inpatient services utilization.

Based on equation (5), the change in the contribution of each variable to the inequality in inpatient services utilization derive from the interaction of a series of change, including the change of the elasticity of corresponding variables and the change of the CI of corresponding variables.

For PCE, higher level change in elasticity rather than in inequality had a dominant role in reducing inequality in probability and frequency of inpatient use (Table 5 and Table 6). It should also be noted that during the period from 2011 to 2015, the unfairness of PCE distribution had intensified (the CI of PCE is equal to 0.0587, 0.0588 and 0.0593, respectively). Therefore, reducing economic disparities between the poor and the rich has important implications for decreasing pro-rich inequality in inpatient services utilization. Relevant departments may take effective measures to improve the economic income and social security level of the poor. Especially for middle-aged and elderly patients with NCDs included in the minimum living guarantee system, more policy benefits should be given.

The lower contribution of UEBMI and NRCMS to the inequality of the probability and number of inpatient use in 2013 compared with 2011 was mainly caused by a certain degree of change of inequality in access to different medical insurance schemes. It indicated that the change of inequality in the coverage of medical insurance schemes during 2011–2013 was the main reason for changing the unfairness of inpatient use. However, Oaxaca-type decomposition also showed that the change of contribution of UEBMI and URCMS in 2015 compared with 2013 was caused by higher level change in elasticity rather than in inequality. A potential explanation for this finding is that there was a “bottleneck” to alleviate inequality in inpatient services utilization by changing inequality in the coverage of medical insurance schemes in the years of 2013 to 2015.

In theory, the decrease in pro-rich inequality of UEBMI and the increase in pro-poor inequality of NRCMS are effective means to reduce the pro-rich inequality of inpatient use for middle-aged and elderly patients with NCDs in China. However, there are several issues that we need to draw attention. Firstly, the coverage rate of basic medical insurance schemes in China exceeded 95% in 2013 [13], and now the “full coverage” of medical insurance schemes has been basically achieved [47]. Secondly, China’s basic medical insurance schemes have been set the scope of insured population in the formulation process, and UEBMI requires the individuals within the scope of the policy must enroll compulsorily [16]. Therefore, current policy system, it has certain limitations to change the inequality of the coverage of medical insurance schemes and improve the coverage rate of medical insurance. To solve these problems, this research deems that breaking through the barriers between different basic medical insurance schemes and resolutely implementing the integration of different medical insurance schemes is a necessary prerequisite to promote the equality in inpatient services utilization. The basic medical insurance schemes should gradually increase the reimbursement rate for NCDs in order to reduce out-of-pocket medical expenditures of residents. In addition, policy makers need to understand that the issue of unfairness in inpatient services utilization cannot depend solely on the efforts of the health sector. In the process of formulating policies, collaboration of multiple departments can minimize the inequality in inpatient services utilization and achieve the goal of improving the health and health equity of the population [48].

There are several limitations in this study. On the one hand, the residual variables made great contribution to the change in inequality of inpatient use, indicating that there are still omitted variables in this study. On the other hand, the indicators of quality or efficiency should be included on fairness research. Unfortunately, the survey did not provide relevant indicators. Therefore, this analysis only referred to the differences in the probability and frequency of inpatient services utilization.

## Conclusion

In conclusion, this study verified that there is a certain degree of pro-rich inequality in the probability and frequency of inpatient services utilization for middle-aged and elderly individuals with NCDs in China. The decrease of pro-wealth contribution of PCE and UEBMI offset the decrease of pro-poor contribution of NRCMS, and improve the equity of inpatient services utilization, favoring poor people.

## Supplementary information

**Additional file 1: Table S1.** Contribution to inequality in the probability of inpatient services utilization, China, 2011. **Table S2.** Contribution to inequality in the probability of inpatient services utilization, China, 2013. **Table S3.** Contribution to inequality in the probability of inpatient services utilization, China, 2015. **Table S4.** Contribution to inequality in the frequency of inpatient services utilization, China, 2011. **Table S5.** Contribution to inequality in the frequency of inpatient services utilization, China, 2013. **Table S6.** Contribution to inequality in the frequency of inpatient services utilization, China, 2015. **Table S7.** Main reason for not seeking inpatient services, China, 2011-2015

## Data Availability

The data source of this study was a publicly available database, the China Health and Retirement Longitudinal Survey (CHARLS), which was hosted by the National School of Development at Peking University (NSD).
