# Supplementary material for: Inequity in inpatient services utilization: a longitudinal comparative analysis of middle-aged and elderly patients with the chronic non-communicable diseases in China
Source: Int J Equity Health. 2020 Jan 6;19:6. doi: 10.1186/s12939-019-1117-9 (PMC6945393; doi:10.1186/s12939-019-1117-9)
Supplement: Supplementary file 1 — Additional file 1: Table S1. Contribution to inequality in the probability of inpatient services utilization, China, 2011. Table S2. Contribution to inequality in the probability of inpatient services utilization, China, 2013. Table S3. Contribution to inequality in the probability of inpatient services utilization, China, 2015. Table S4. Contribution to inequality in the frequency of inpatient services utilization, China, 2011. Table S5. Contribution to inequality in the frequency of inpatient services utilization, China, 2013. Table S6. Contribution to inequality in the frequency of inpatient services utilization, China, 2015. Table S7. Main reason for not seeking inpatient services, China, 2011-2015 [file 12939_2019_1117_MOESM1_ESM.docx]

**Supplemental Table I.** Contribution to inequality in the probability of inpatient services utilization, China, 2011

| Variable | Marginal effect | Std. Err. | Elasticity | CI | Contribution | Percent |
| --- | --- | --- | --- | --- | --- | --- |
| Predisposing variables |  |  |  |  |  |  |
| Gender |  |  |  |  |  |  |
| Female^1^ |  |  |  |  |  |  |
| Male | 0.0201** | 0.0064 | 0.0800 | 0.0125 | 0.0010 | 0.6768 |
| Age |  |  |  |  |  |  |
| 45~59^1^ |  |  |  |  |  |  |
| 60~74 | 0.0281** | 0.0070 | 0.0913 | -0.0380 | -0.0035 | -2.3466 |
| 75+ | 0.0507** | 0.0143 | 0.0388 | -0.0890 | -0.0035 | -2.3334 |
| Education level |  |  |  |  |  |  |
| Illiterate^1^ |  |  |  |  |  |  |
| Primary school | -0.0108 | 0.0076 | -0.0196 | -0.0186 | 0.0004 | 0.2461 |
| Middle school | -0.0001 | 0.0088 | -0.0002 | 0.1221 | -0.00002 | -0.0146 |
| High school and above | -0.0143 | 0.0106 | -0.0139 | 0.3354 | -0.0047 | -3.1486 |
| Marital status |  |  |  |  |  |  |
| Unmarried^1^ |  |  |  |  |  |  |
| Married | 0.0229** | 0.0080 | 0.1659 | 0.0114 | 0.0019 | 1.2842 |
| Employment status |  |  |  |  |  |  |
| Unemployed^1^ |  |  |  |  |  |  |
| Agricultural work | -0.0287** | 0.0070 | -0.0895 | -0.1590 | 0.0142 | 9.6222 |
| Working in units | -0.0448** | 0.0083 | -0.0497 | 0.1394 | -0.0069 | -4.6804 |
| Other working | -0.0503** | 0.0090 | -0.0340 | 0.1310 | -0.0044 | -3.0071 |
| Enabling variables |  |  |  |  |  |  |
| Health insurance schemes |  |  |  |  |  |  |
| No health insurance^1^ |  |  |  |  |  |  |
| UEBMI | 0.1088** | 0.0261 | 0.0921 | 0.4693 | 0.0432 | 29.2131 |
| URBMI | 0.0833** | 0.0285 | 0.0312 | 0.2159 | 0.0067 | 4.5482 |
| NRCMS | 0.0599** | 0.0119 | 0.3591 | -0.0998 | -0.0359 | -24.2418 |
| Other health insurance | 0.0992** | 0.0269 | 0.0591 | 0.2532 | 0.0150 | 10.1228 |
| Geographic location |  |  |  |  |  |  |
| East^1^ |  |  |  |  |  |  |
| Central | 0.0212** | 0.0080 | 0.0610 | -0.0345 | -0.0021 | -1.4252 |
| West | 0.0463** | 0.0082 | 0.1344 | -0.0342 | -0.0046 | -3.1039 |
| Residency location |  |  |  |  |  |  |
| Rural^1^ |  |  |  |  |  |  |
| Urban | 0.0111 | 0.0073 | 0.0363 | 0.2123 | 0.0077 | 5.2094 |
| PCE | 0.0339** | 0.0035 | 2.4154 | 0.0587 | 0.1419 | 95.9444 |
| Need variables |  |  |  |  |  |  |
| Self-assessed health status |  |  |  |  |  |  |
| Unhealthy^1^ |  |  |  |  |  |  |
| Healthy | -0.0849** | 0.0071 | -0.4366 | 0.0309 | -0.0135 | -9.1275 |
| Disability |  |  |  |  |  |  |
| No^1^ |  |  |  |  |  |  |
| Yes | -0.0024 | 0.0073 | -0.0042 | -0.1152 | 0.0005 | 0.3289 |
| PADL |  |  |  |  |  |  |
| No^1^ |  |  |  |  |  |  |
| Yes | 0.0212** | 0.0084 | 0.0376 | -0.0903 | -0.0034 | -2.2943 |
| IADL |  |  |  |  |  |  |
| No^1^ |  |  |  |  |  |  |
| Yes | 0.0323** | 0.0084 | 0.0701 | -0.0934 | -0.0065 | -4.4248 |
| Residual variables |  |  |  |  | 0.0044 | 2.9750 |

Note: * p < 0.05; ** p < 0.01; ^1^ Reference group; ^2^ Marginal effect; ^3^ Contribution; PCE = logarithm value of the per capita household expenditure; PADL = Physical Activity of Daily Living; IADL = Instrumental Activity of Daily Living; UEBMI = Urban Employee Basic Medical Insurance; NRCMS = New Rural Cooperative Medical Scheme; URBMI = Urban Residents Basic Medical Insurance.

**Supplemental Table II.** Contribution to inequality in the probability of inpatient services utilization, China, 2013

| Variable | Marginal effect | Std. Err. | Elasticity | CI | Contribution | Percent |
| --- | --- | --- | --- | --- | --- | --- |
| Predisposing variables |  |  |  |  |  |  |
| Gender |  |  |  |  |  |  |
| Female^1^ |  |  |  |  |  |  |
| Male | 0.0257** | 0.0087 | 0.0732 | 0.0158 | 0.0012 | 0.9428 |
| Age |  |  |  |  |  |  |
| 45~59^1^ |  |  |  |  |  |  |
| 60~74 | 0.0354** | 0.0094 | 0.0978 | -0.0491 | -0.0048 | -3.9123 |
| 75+ | 0.0666** | 0.0193 | 0.0360 | -0.0902 | -0.0032 | -2.6454 |
| Education level |  |  |  |  |  |  |
| Illiterate^1^ |  |  |  |  |  |  |
| Primary school | -0.0015 | 0.0106 | -0.0020 | -0.0097 | 0.00002 | 0.0158 |
| Middle school | -0.0177 | 0.0114 | -0.0220 | 0.1010 | -0.0022 | -1.8122 |
| High school and above | -0.0100 | 0.0147 | -0.0071 | 0.2899 | -0.0021 | -1.6748 |
| Marital status |  |  |  |  |  |  |
| Unmarried^1^ |  |  |  |  |  |  |
| Married | 0.0043 | 0.0121 | 0.0229 | 0.0097 | 0.0002 | 0.1801 |
| Employment status |  |  |  |  |  |  |
| Unemployed^1^ |  |  |  |  |  |  |
| Agricultural work | -0.0325** | 0.0097 | -0.0846 | -0.0914 | 0.0077 | 6.2974 |
| Working in units | -0.0418** | 0.0130 | -0.0290 | 0.0823 | -0.0024 | -1.9475 |
| Other working | -0.0396** | 0.0138 | -0.0204 | 0.1165 | -0.0024 | -1.9376 |
| Enabling variables |  |  |  |  |  |  |
| Health insurance schemes |  |  |  |  |  |  |
| No health insurance^1^ |  |  |  |  |  |  |
| UEBMI | 0.0923** | 0.0382 | 0.0648 | 0.3284 | 0.0213 | 17.3403 |
| URBMI | 0.0005 | 0.0334 | 0.0001 | 0.1213 | 0.00002 | 0.0147 |
| NRCMS | 0.0583* | 0.0232 | 0.2633 | -0.0758 | -0.0200 | -16.2541 |
| Other health insurance | 0.0746* | 0.0384 | 0.0327 | 0.1960 | 0.0064 | 5.2263 |
| Geographic location |  |  |  |  |  |  |
| East^1^ |  |  |  |  |  |  |
| Central | 0.0356** | 0.0110 | 0.0717 | -0.0225 | -0.0016 | -1.3126 |
| West | 0.0604** | 0.0107 | 0.1350 | 0.0032 | 0.0004 | 0.3535 |
| Residency location |  |  |  |  |  |  |
| Rural^1^ |  |  |  |  |  |  |
| Urban | 0.0220* | 0.0100 | 0.0514 | 0.1394 | 0.0072 | 5.8396 |
| PCE | 0.0435** | 0.0044 | 2.3793 | 0.0588 | 0.1399 | 113.8950 |
| Need variables |  |  |  |  |  |  |
| Self-assessed health status |  |  |  |  |  |  |
| Unhealthy^1^ |  |  |  |  |  |  |
| Healthy | -0.1443** | 0.0100 | -0.5836 | 0.0249 | -0.0145 | -11.8197 |
| Disability |  |  |  |  |  |  |
| No^1^ |  |  |  |  |  |  |
| Yes | 0.0161 | 0.0092 | 0.0289 | -0.0575 | -0.0017 | -1.3528 |
| PADL |  |  |  |  |  |  |
| No^1^ |  |  |  |  |  |  |
| Yes | 0.0106 | 0.0109 | 0.0135 | -0.0666 | -0.0009 | -0.7320 |
| IADL |  |  |  |  |  |  |
| No^1^ |  |  |  |  |  |  |
| Yes | 0.0186 | 0.0109 | 0.0264 | -0.0631 | -0.0017 | -1.3584 |
| Residual variables |  |  |  |  | -0.0040 | -3.2573 |

Note: * p < 0.05; ** p < 0.01; ^1^ Reference group; ^2^ Marginal effect; ^3^ Contribution; PCE = logarithm value of the per capita household expenditure; PADL = Physical Activity of Daily Living; IADL = Instrumental Activity of Daily Living; UEBMI = Urban Employee Basic Medical Insurance; NRCMS = New Rural Cooperative Medical Scheme; URBMI = Urban Residents Basic Medical Insurance.

**Supplemental Table III.** Contribution to inequality in the probability of inpatient services utilization, China, 2015

| Variable | Marginal effect | Std. Err. | Elasticity | CI | Contribution | Percent |
| --- | --- | --- | --- | --- | --- | --- |
| Predisposing variables |  |  |  |  |  |  |
| Gender |  |  |  |  |  |  |
| Female^1^ |  |  |  |  |  |  |
| Male | 0.0061 | 0.0078 | 0.0166 | 0.0184 | 0.0003 | 0.3134 |
| Age |  |  |  |  |  |  |
| 45~59^1^ |  |  |  |  |  |  |
| 60~74 | 0.0372** | 0.0084 | 0.1032 | -0.0456 | -0.0047 | -4.8040 |
| 75+ | 0.0671** | 0.0166 | 0.0365 | -0.1209 | -0.0044 | -4.5028 |
| Education level |  |  |  |  |  |  |
| Illiterate^1^ |  |  |  |  |  |  |
| Primary school | 0.0077 | 0.0097 | 0.0101 | -0.0185 | -0.0002 | -0.1903 |
| Middle school | 0.0190 | 0.0110 | 0.0226 | 0.0698 | 0.0016 | 1.6085 |
| High school and above | 0.0000 | 0.0136 | 0.0000 | 0.2967 | 0.0000 | 0.0000 |
| Marital status |  |  |  |  |  |  |
| Unmarried^1^ |  |  |  |  |  |  |
| Married | 0.0019 | 0.0105 | 0.0093 | 0.0070 | 0.0001 | 0.0666 |
| Employment status |  |  |  |  |  |  |
| Unemployed^1^ |  |  |  |  |  |  |
| Agricultural work | -0.0495** | 0.0084 | -0.1055 | -0.1017 | 0.0107 | 10.9609 |
| Working in units | -0.0587** | 0.0117 | -0.0306 | 0.1346 | -0.0041 | -4.2031 |
| Other working | -0.0525** | 0.0102 | -0.0465 | 0.0518 | -0.0024 | -2.4587 |
| Enabling variables |  |  |  |  |  |  |
| Health insurance schemes |  |  |  |  |  |  |
| No health insurance^1^ |  |  |  |  |  |  |
| UEBMI | 0.0849** | 0.0235 | 0.0491 | 0.3356 | 0.0164 | 16.8262 |
| URBMI | 0.0466 | 0.0266 | 0.0111 | 0.0797 | 0.0009 | 0.9020 |
| NRCMS | 0.0402** | 0.0141 | 0.1530 | -0.0789 | -0.0121 | -12.3312 |
| Other health insurance | 0.0647** | 0.0205 | 0.0499 | 0.1426 | 0.0071 | 7.2683 |
| Geographic location |  |  |  |  |  |  |
| East^1^ |  |  |  |  |  |  |
| Central | 0.0166 | 0.0094 | 0.0319 | -0.0185 | -0.0006 | -0.6032 |
| West | 0.0501** | 0.0094 | 0.1014 | 0.0050 | 0.0005 | 0.5191 |
| Residency location |  |  |  |  |  |  |
| Rural^1^ |  |  |  |  |  |  |
| Urban | 0.0062 | 0.0087 | 0.0141 | 0.1209 | 0.0017 | 1.7374 |
| PCE | 0.0320** | 0.0038 | 1.6988 | 0.0593 | 0.1008 | 102.9577 |
| Need variables |  |  |  |  |  |  |
| Self-assessed health status |  |  |  |  |  |  |
| Unhealthy^1^ |  |  |  |  |  |  |
| Healthy | -0.1381** | 0.0090 | -0.5317 | 0.0186 | -0.0099 | -10.1004 |
| Disability |  |  |  |  |  |  |
| No^1^ |  |  |  |  |  |  |
| Yes | 0.0123 | 0.0078 | 0.0262 | -0.0752 | -0.0020 | -2.0088 |
| PADL |  |  |  |  |  |  |
| No^1^ |  |  |  |  |  |  |
| Yes | 0.0494** | 0.0099 | 0.0693 | -0.0627 | -0.0043 | -4.4357 |
| IADL |  |  |  |  |  |  |
| No^1^ |  |  |  |  |  |  |
| Yes | 0.0304** | 0.0096 | 0.0451 | -0.0602 | -0.0027 | -2.7749 |
| Residual variables |  |  |  |  | 0.0052 | 5.3115 |

Note: * p < 0.05; ** p < 0.01; ^1^ Reference group; ^2^ Marginal effect; ^3^ Contribution; PCE = logarithm value of the per capita household expenditure; PADL = Physical Activity of Daily Living; IADL = Instrumental Activity of Daily Living; UEBMI = Urban Employee Basic Medical Insurance; NRCMS = New Rural Cooperative Medical Scheme; URBMI = Urban Residents Basic Medical Insurance.

**Supplemental Table IV.** Contribution to inequality in the frequency of inpatient services utilization, China, 2011

| Variable | Marginal effect | Std. Err. | Elasticity | CI | Contribution | Percent |
| --- | --- | --- | --- | --- | --- | --- |
| Predisposing variables |  |  |  |  |  |  |
| Gender |  |  |  |  |  |  |
| Female^1^ |  |  |  |  |  |  |
| Male | 0.0428** | 0.0134 | 0.1178 | 0.0125 | 0.0015 | 0.8687 |
| Age |  |  |  |  |  |  |
| 45~59^1^ |  |  |  |  |  |  |
| 60~74 | 0.0492** | 0.0140 | 0.1105 | -0.0380 | -0.0042 | -2.4788 |
| 75+ | 0.0916** | 0.0228 | 0.0484 | -0.0890 | -0.0043 | -2.5417 |
| Education level |  |  |  |  |  |  |
| Illiterate^1^ |  |  |  |  |  |  |
| Primary school | -0.0229 | 0.0171 | -0.0287 | -0.0186 | 0.0005 | 0.3151 |
| Middle school | -0.0142 | 0.0167 | -0.0161 | 0.1220 | -0.0020 | -1.1594 |
| High school and above | -0.0290 | 0.0228 | -0.0195 | 0.3354 | -0.0065 | -3.8624 |
| Marital status |  |  |  |  |  |  |
| Unmarried^1^ |  |  |  |  |  |  |
| Married | 0.0338* | 0.0175 | 0.1693 | 0.0114 | 0.0019 | 1.1432 |
| Employment status |  |  |  |  |  |  |
| Unemployed^1^ |  |  |  |  |  |  |
| Agricultural work | -0.0630** | 0.0150 | -0.1362 | -0.1590 | 0.0216 | 12.7722 |
| Working in units | -0.1070** | 0.0222 | -0.0821 | 0.1394 | -0.0114 | -6.7503 |
| Other working | -0.0999** | 0.0282 | -0.0467 | 0.1309 | -0.0061 | -3.6078 |
| Enabling variables |  |  |  |  |  |  |
| Health insurance schemes |  |  |  |  |  |  |
| No health insurance^1^ |  |  |  |  |  |  |
| UEBMI | 0.1202** | 0.0410 | 0.0704 | 0.4693 | 0.0330 | 19.4919 |
| URBMI | 0.0902* | 0.0437 | 0.0233 | 0.2158 | 0.0050 | 2.9725 |
| NRCMS | 0.0875* | 0.0391 | 0.3630 | -0.0998 | -0.0362 | -21.3832 |
| Other health insurance | 0.1204** | 0.0428 | 0.0496 | 0.2532 | 0.0126 | 7.4161 |
| Geographic location |  |  |  |  |  |  |
| East^1^ |  |  |  |  |  |  |
| Central | 0.0465** | 0.0155 | 0.0924 | -0.0345 | -0.0032 | -1.8830 |
| West | 0.0899** | 0.0153 | 0.1806 | -0.0342 | -0.0062 | -3.6405 |
| Residency location |  |  |  |  |  |  |
| Rural^1^ |  |  |  |  |  |  |
| Urban | 0.0064 | 0.0148 | 0.0145 | 0.2123 | 0.0031 | 1.8167 |
| PCE | 0.0647** | 0.0075 | 3.1893 | 0.0587 | 0.1873 | 110.5308 |
| Need variables |  |  |  |  |  |  |
| Self-assessed health status |  |  |  |  |  |  |
| Unhealthy^1^ |  |  |  |  |  |  |
| Healthy | -0.1537** | 0.0142 | -0.5471 | 0.0309 | -0.0169 | -9.9804 |
| Disability |  |  |  |  |  |  |
| No^1^ |  |  |  |  |  |  |
| Yes | -0.0082 | 0.0144 | -0.0098 | -0.1152 | 0.0011 | 0.6654 |
| PADL |  |  |  |  |  |  |
| No^1^ |  |  |  |  |  |  |
| Yes | 0.0375** | 0.0137 | 0.0461 | -0.0903 | -0.0042 | -2.4540 |
| IADL |  |  |  |  |  |  |
| No^1^ |  |  |  |  |  |  |
| Yes | 0.0534** | 0.0132 | 0.0802 | -0.0934 | -0.0075 | -4.4183 |
| Residual variables |  |  |  |  | 0.0106 | 6.2537 |

Note: * p < 0.05; ** p < 0.01; ^1^ Reference group; ^2^ Marginal effect; ^3^ Contribution; PCE = logarithm value of the per capita household expenditure; PADL = Physical Activity of Daily Living; IADL = Instrumental Activity of Daily Living; UEBMI = Urban Employee Basic Medical Insurance; NRCMS = New Rural Cooperative Medical Scheme; URBMI = Urban Residents Basic Medical Insurance.

**Supplemental Table V.** Contribution to inequality in the frequency of inpatient services utilization, China, 2013

| Variable | Marginal effect | Std. Err. | Elasticity | CI | Contribution | Percent |
| --- | --- | --- | --- | --- | --- | --- |
| Predisposing variables |  |  |  |  |  |  |
| Gender |  |  |  |  |  |  |
| Female^1^ |  |  |  |  |  |  |
| Male | 0.0581** | 0.0187 | 0.1068 | 0.0158 | 0.0017 | 1.2137 |
| Age |  |  |  |  |  |  |
| 45~59^1^ |  |  |  |  |  |  |
| 60~74 | 0.0536** | 0.0200 | 0.0956 | -0.0491 | -0.0047 | -3.3739 |
| 75+ | 0.1075** | 0.0323 | 0.0374 | -0.0902 | -0.0034 | -2.4277 |
| Education level |  |  |  |  |  |  |
| Illiterate^1^ |  |  |  |  |  |  |
| Primary school | -0.0016 | 0.0226 | -0.0014 | -0.0097 | 0.0000 | 0.0095 |
| Middle school | -0.0470 | 0.0249 | -0.0377 | 0.1010 | -0.0038 | -2.7343 |
| High school and above | -0.0914** | 0.0298 | -0.0419 | 0.2899 | -0.0121 | -8.7309 |
| Marital status |  |  |  |  |  |  |
| Unmarried^1^ |  |  |  |  |  |  |
| Married | 0.0047 | 0.0251 | 0.0163 | 0.0097 | 0.0002 | 0.1133 |
| Employment status |  |  |  |  |  |  |
| Unemployed^1^ |  |  |  |  |  |  |
| Agricultural work | -0.0980** | 0.0209 | -0.1641 | -0.0914 | 0.0150 | 10.7847 |
| Working in units | -0.1191** | 0.0341 | -0.0533 | 0.0823 | -0.0044 | -3.1564 |
| Other working | -0.0849** | 0.0410 | -0.0282 | 0.1165 | -0.0033 | -2.3602 |
| Enabling variables |  |  |  |  |  |  |
| Health insurance schemes |  |  |  |  |  |  |
| No health insurance^1^ |  |  |  |  |  |  |
| UEBMI | 0.1721** | 0.0602 | 0.0779 | 0.3283 | 0.0256 | 18.3869 |
| URBMI | 0.0693 | 0.0699 | 0.0135 | 0.1213 | 0.0016 | 1.1801 |
| NRCMS | 0.1433** | 0.0549 | 0.4171 | -0.0758 | -0.0316 | -22.7286 |
| Other health insurance | 0.1625** | 0.0617 | 0.0459 | 0.1960 | 0.0090 | 6.4703 |
| Geographic location |  |  |  |  |  |  |
| East^1^ |  |  |  |  |  |  |
| Central | 0.0974** | 0.0221 | 0.1265 | -0.0225 | -0.0028 | -2.0429 |
| West | 0.1370** | 0.0212 | 0.1972 | 0.0032 | 0.0006 | 0.4549 |
| Residency location |  |  |  |  |  |  |
| Rural^1^ |  |  |  |  |  |  |
| Urban | 0.0299 | 0.0203 | 0.0450 | 0.1393 | 0.0063 | 4.5123 |
| PCE | 0.0816** | 0.0098 | 2.8718 | 0.0588 | 0.1688 | 121.3475 |
| Need variables |  |  |  |  |  |  |
| Self-assessed health status |  |  |  |  |  |  |
| Unhealthy^1^ |  |  |  |  |  |  |
| Healthy | -0.2319** | 0.0192 | -0.6040 | 0.0249 | -0.0150 | -10.8013 |
| Disability |  |  |  |  |  |  |
| No^1^ |  |  |  |  |  |  |
| Yes | 0.0309 | 0.0183 | 0.0356 | -0.0575 | -0.0021 | -1.4741 |
| PADL |  |  |  |  |  |  |
| No^1^ |  |  |  |  |  |  |
| Yes | 0.0339 | 0.0199 | 0.0279 | -0.0666 | -0.0019 | -1.3354 |
| IADL |  |  |  |  |  |  |
| No^1^ |  |  |  |  |  |  |
| Yes | 0.0276 | 0.0193 | 0.0253 | -0.0631 | -0.0016 | -1.1469 |
| Residual variables |  |  |  |  | -0.0030 | -2.1567 |

Note: * p < 0.05; ** p < 0.01; ^1^ Reference group; ^2^ Marginal effect; ^3^ Contribution; PCE = logarithm value of the per capita household expenditure; PADL = Physical Activity of Daily Living; IADL = Instrumental Activity of Daily Living; UEBMI = Urban Employee Basic Medical Insurance; NRCMS = New Rural Cooperative Medical Scheme; URBMI = Urban Residents Basic Medical Insurance.

**Supplemental Table VI.** Contribution to inequality in the frequency of inpatient services utilization, China, 2015

| Variable | Marginal effect | Std. Err. | Elasticity | CI | Contribution | Percent |
| --- | --- | --- | --- | --- | --- | --- |
| Predisposing variables |  |  |  |  |  |  |
| Gender |  |  |  |  |  |  |
| Female^1^ |  |  |  |  |  |  |
| Male | 0.0058 | 0.0150 | 0.0103 | 0.0184 | 0.0002 | 0.1599 |
| Age |  |  |  |  |  |  |
| 45~59^1^ |  |  |  |  |  |  |
| 60~74 | 0.0643** | 0.0167 | 0.1164 | -0.0456 | -0.0053 | -4.4753 |
| 75+ | 0.1316** | 0.0272 | 0.0467 | -0.1209 | -0.0056 | -4.7626 |
| Education level |  |  |  |  |  |  |
| Illiterate^1^ |  |  |  |  |  |  |
| Primary school | 0.0039 | 0.0180 | 0.0033 | -0.0185 | -0.0001 | -0.0517 |
| Middle school | 0.0280 | 0.0199 | 0.0217 | 0.0698 | 0.0015 | 1.2758 |
| High school and above | -0.0172 | 0.0243 | -0.0080 | 0.2967 | -0.0024 | -1.9960 |
| Marital status |  |  |  |  |  |  |
| Unmarried^1^ |  |  |  |  |  |  |
| Married | 0.0181 | 0.0207 | 0.0594 | 0.0070 | 0.0004 | 0.3508 |
| Employment status |  |  |  |  |  |  |
| Unemployed^1^ |  |  |  |  |  |  |
| Agricultural work | -0.1035** | 0.0181 | -0.1438 | -0.1017 | 0.0146 | 12.3430 |
| Working in units | -0.1717** | 0.0316 | -0.0583 | 0.1346 | -0.0078 | -6.6213 |
| Other working | -0.1189** | 0.0245 | -0.0687 | 0.0518 | -0.0036 | -2.9994 |
| Enabling variables |  |  |  |  |  |  |
| Health insurance schemes |  |  |  |  |  |  |
| No health insurance^1^ |  |  |  |  |  |  |
| UEBMI | 0.1115** | 0.0369 | 0.0420 | 0.3356 | 0.0141 | 11.9059 |
| URBMI | 0.0765 | 0.0445 | 0.0119 | 0.0797 | 0.0009 | 0.7986 |
| NRCMS | 0.0475 | 0.0304 | 0.1178 | -0.0789 | -0.0093 | -7.8450 |
| Other health insurance | 0.1076** | 0.0344 | 0.0541 | 0.1426 | 0.0077 | 6.5155 |
| Geographic location |  |  |  |  |  |  |
| East^1^ |  |  |  |  |  |  |
| Central | 0.0322 | 0.0184 | 0.0403 | -0.0185 | -0.0007 | -0.6301 |
| West | 0.0917** | 0.0186 | 0.1210 | 0.0050 | 0.0006 | 0.5135 |
| Residency location |  |  |  |  |  |  |
| Rural^1^ |  |  |  |  |  |  |
| Urban | 0.0013 | 0.0171 | 0.0019 | 0.1209 | 0.0002 | 0.1957 |
| PCE | 0.0605** | 0.0083 | 2.0972 | 0.0593 | 0.1244 | 104.9972 |
| Need variables |  |  |  |  |  |  |
| Self-assessed health status |  |  |  |  |  |  |
| Unhealthy^1^ |  |  |  |  |  |  |
| Healthy | -0.2533** | 0.0175 | -0.6360 | 0.0186 | -0.0118 | -9.9860 |
| Disability |  |  |  |  |  |  |
| No^1^ |  |  |  |  |  |  |
| Yes | 0.0253 | 0.0159 | 0.0351 | -0.0752 | -0.0026 | -2.2237 |
| PADL |  |  |  |  |  |  |
| No^1^ |  |  |  |  |  |  |
| Yes | 0.0933** | 0.0192 | 0.0854 | -0.0627 | -0.0054 | -4.5154 |
| IADL |  |  |  |  |  |  |
| No^1^ |  |  |  |  |  |  |
| Yes | 0.0499** | 0.0192 | 0.0484 | -0.0602 | -0.0029 | -2.4581 |
| Residual variables |  |  |  |  | 0.0113 | 9.5087 |

Note: * p < 0.05; ** p < 0.01; ^1^ Reference group; ^2^ Marginal effect; ^3^ Contribution; PCE = logarithm value of the per capita household expenditure; PADL = Physical Activity of Daily Living; IADL = Instrumental Activity of Daily Living; UEBMI = Urban Employee Basic Medical Insurance; NRCMS = New Rural Cooperative Medical Scheme; URBMI = Urban Residents Basic Medical Insurance.

**Supplemental Table VII.** Main reason for not seeking inpatient services, China, 2011-2015.

|  | 2011 | 2013 | 2015 |
| --- | --- | --- | --- |
| The main reasons for not seeking hospitalization(%) |  |  |  |
| Not enough money | 62.81 | 55.97 | 60.00 |
| Not willing to go to the hospital | 20.72 | 21.64 | 20.00 |
| Hospital quality poor | 2.28 | 2.80 | 4.21 |
| Problem too serious | 1.79 | 4.10 | 2.11 |
| No ward available | 0.65 | 1.49 | 1.05 |
| Other | 11.75 | 13.99 | 12.63 |
| The concentration index of the main reasons for not seeking hospitalization |  |  |  |
| Not enough money | -0.0754 | -0.0540 | -0.0717 |
| Not willing to go to the hospital | 0.1548 | 0.0318 | -0.0097 |
| Hospital quality poor | 0.1237 | 0.1864 | 0.3086 |
| Problem too serious | 0.2106 | 0.0675 | 0.3144 |
| No ward available | 0.4127 | 0.1931 | 0.0023 |
| Other | 0.0508 | 0.0889 | 0.1231 |
| Out-of-pocket inpatient expenditure as a percentage of total inpatient expenditure (%) |  |  |  |
| Patients enrolled in UEBMI | 28.33 | 34.90 | 30.95 |
| Patients enrolled in URBMI | 61.42 | 40.92 | 50.91 |
| Patients enrolled in NRCMS | 72.71 | 57.36 | 65.77 |

Note: UEBMI = Urban Employee Basic Medical Insurance; NRCMS = New Rural Cooperative Medical Scheme; URBMI = Urban Residents Basic Medical Insurance.
